# MicroRNAs as immune regulators and biomarkers in tuberculosis

**DOI:** 10.3389/fimmu.2022.1027472

**Published:** 2022-10-27

**Authors:** Lulu Wang, Yan Xiong, Beibei Fu, Dong Guo, Mohamed Y. Zaky, Xiaoyuan Lin, Haibo Wu

**Affiliations:** ^1^ Department of Biology, School of Life Sciences, Chongqing University, Chongqing, China; ^2^ Department of Zoology, Molecular Physiology Division, Faculty of Science, Beni-Suef University, Beni-Suef, Egypt

**Keywords:** microRNA, *Mycobacterium tuberculosis*, host immunity, immune regulators, biomarkers

## Abstract

Tuberculosis (TB), which is caused by *Mycobacterium tuberculosis* (Mtb), is one of the most lethal infectious disease worldwide, and it greatly affects human health. Some diagnostic and therapeutic methods are available to effectively prevent and treat TB; however, only a few systematic studies have described the roles of microRNAs (miRNAs) in TB. Combining multiple clinical datasets and previous studies on Mtb and miRNAs, we state that pathogens can exploit interactions between miRNAs and other biomolecules to avoid host mechanisms of immune-mediated clearance and survive in host cells for a long time. During the interaction between Mtb and host cells, miRNA expression levels are altered, resulting in the changes in the miRNA-mediated regulation of host cell metabolism, inflammatory responses, apoptosis, and autophagy. In addition, differential miRNA expression can be used to distinguish healthy individuals, patients with TB, and patients with latent TB. This review summarizes the roles of miRNAs in immune regulation and their application as biomarkers in TB. These findings could provide new opportunities for the diagnosis and treatment of TB.

## Introduction

Tuberculosis (TB), which is the 13th leading cause of death worldwide, is an infectious disease that seriously threatens human health. According to the World Health Organization *Global Tuberculosis Report 2021*, 1.5 million people worldwide died of TB in 2020, making it the second most lethal infectious disease after coronavirus disease 2019 (COVID-19) (1). Despite the devastating effects of TB, TB research has been limited by the COVID-19 pandemic, and conflicts in Europe, Africa, and the Middle East have caused disruptions in essential TB services and increased TB-related deaths, making TB one of the most lethal infectious diseases in the world.

*Mycobacterium tuberculosis* (Mtb) is the pathogen that causes TB. Mtb is an obligate aerobic bacterium that is characterized by positive acid-fast staining (2). Although it possesses fimbriae and microcapsules, Mtb does not form spores. In addition, its bacterial wall contains neither the teichoic acid of gram-positive bacteria nor the lipopolysaccharide of gram-negative bacteria (3). Furthermore, Mtb has approximately 4000 genes with high guanine and cytosine contents (4). During infection, Mtb causes inflammatory responses and immune-mediated damage to the host (5). The main pathogenic substances of Mtb include capsules, lipids, and proteins (6). Additionally, although 2-7 h are required to kill Mtb in sputum with direct sunlight, Mtb is very resistant to dry, cold, acidic, and alkaline conditions. Therefore, Mtb can invade all tissues and organs of the body, with the most common site of infection being the lungs. Macrophages are the primary host cells of Mtb and the primary immune cells that function to clear Mtb. Macrophages eliminate Mtb by inducing apoptosis, autophagy, and inflammatory responses, which are critical for innate immunity (7).

However, due to the dynamic nature of Mtb, it can interact with host cells to induce conditions that are more conducive to its survival. For example, Mtb alters the expression of host microRNAs (miRNAs) to regulate the expression of immune-related genes and promote its long-term survival. Furthermore, Mtb not only inhibits the clearance mechanisms of the host but also remains in host cells, ultimately causing latent tuberculosis infection (LTBI) (6). It is also estimated that one-quarter of the global population has LTBI, and of these patients, 5%–10% are at risk of developing active TB. As a result, failure to effectively control LTBI threatens the achievement of “End TB” goals. Antibiotics are the gold standard treatment for TB. However, although antibiotics (isoniazid, rifampicin, and streptomycin) are effective in the treatment of TB (8), Mtb can acquire drug-resistant phenotypes. Drug-resistant TB is simultaneously resistant to multiple drugs, meaning that multiple drugs have no therapeutic effect on TB (9). Thus, the increasing number of patients with drug-resistant disease each year complicates TB treatment. Mtb also uses immune evasion mechanisms to remain latent in host cells for a long time. Notably, previous studies have shown that Mtb can survive inside cells by regulating the miRNA expression of host cells (10, 11).

miRNAs are a class of small noncoding RNAs that are approximately 20-24 nucleotides in length. Although they do not encode proteins, they are notably involved in regulating gene expression at the posttranscriptional level (10). miRNAs also inhibit gene expression by targeting the translation of specific mRNAs (12). Additionally, miRNAs play regulatory roles in many important physiological processes, such as cell proliferation and differentiation, body metabolism, and host immunity (13, 14). Infection with certain pathogens affect miRNA expression in the host, and miRNAs may play instrumental roles in directing immune responses (15). Moreover, in Mtb-infected cells, miRNAs appear to be used by Mtb to modulate host immunity (16). It has also been reported that the specific expression patterns of miRNAs can be used as potential diagnostic biomarkers in TB (17).

Therefore, this paper mainly discusses the regulatory roles of miRNAs in metabolism, inflammatory responses, autophagy, and apoptosis during Mtb infection. We also summarize advances in the use of miRNAs as biomarkers in TB and further discuss the promises and challenges associated with their use as biomarkers.

## Regulation of host cell metabolism and inflammatory factors by miRNAs

### miR-21 regulates host glycolysis

Glycolysis is a common process of glucose catabolism that occurs in almost all biological cells and can occur under both aerobic and anaerobic conditions (18). Glycolysis involves the transformation of glucose into pyruvate through several enzymatic steps, simultaneously yielding adenosine triphosphate (ATP) and nicotinamide adenine dinucleotide. Glycolysis is the most critical process of glucose metabolism, and it the first metabolic pathway to be elucidated (19).

When Mtb infects a host, glycolysis is a major metabolic process that promotes inflammatory responses in immune cells (20). To date, most studies on the immune-metabolic effects of Mtb infection have been conducted in macrophages. Efferocytosis is the process by which macrophages engulf and eliminate apoptotic cells, and it is used by the host to control infection when macrophages are exposed to pathogens (21). After Mtb infection, uninfected macrophages can phagocytose infected macrophages through efferocytosis (22). Based on these studies, Mtb infections result in poor innate immunity of macrophages, contributing to pathogen survival (23). Several authors have also shown increased activation of metabolic pathways in Mtb-infected macrophages (24–26). Other studies have shown that continuous glycolysis supports the production of the proinflammatory cytokine interleukin (IL)-1β, which, in turn, regulates prostaglandin 2 to control Mtb infection (27). After pathogen invasion, macrophages change their metabolic profile from oxidative respiration to high-rate aerobic glycolysis through the tricarboxylic acid cycle. This immunometabolic shift supports the production of the proinflammatory cytokine interleukin (IL)-1β, which promotes proinflammatory and antibacterial responses (28).

miR-21 regulates intracellular glycolysis and limits macrophage metabolic reprogramming during Mtb infection. In a previous study, measurements of the extracellular acidification rate suggested that anti-miR-21 can substantially improve the glycolysis and glycolytic capacity of rat cardiomyoblast cells (29). Another study has proven that miR-21 impairs antimycobacterial responses by targeting IL-12 and B-cell lymphoma/leukemia-2 (Bcl-2) (30). It has also been shown that Mtb inhibits phosphor-fructokinase, muscle (PFK-M) *via* miR-21 to limit glycolysis in host cells (31). Lung tissues of mice infected with Mtb upregulate pri-miR-21 30 days after infection and maintain high levels of pri-miR-21 for 53 days. When murine bone marrow-derived macrophages (BMDMs) are infected with Mtb, miR-21 is continuously upregulated for 72 h and targets PFK-M at the critical step of glycolysis to inhibit this process. For the host, interferon-γ (IFN-γ), which drives host defenses against Mtb, inhibits miR-21 expression, forcing an isoenzyme switch in the PFK complex and maintaining PFK-M expression after Mtb infection. Therefore, miR-21 targets PFK-M to control macrophage immunometabolic function (31).

### miR-33 regulates liposome catabolism

Lipids form fundamental components of cell membranes and are necessary for many physiological functions, such as energy supply, signal transduction, and cell recognition (32). Lipid metabolism refers to the synthesis, decomposition, digestion, and absorption of lipids by various enzymes. It also involves the processing of lipids into substances that are needed to maintain activities related to biological homeostasis. Evidence has shown that abnormal lipid metabolism can cause changes in membrane composition and permeability, resulting in the occurrence and development of various diseases (33). Simultaneously, specific changes in lipid synthesis and metabolism occur during pathogen invasion and carcinogenesis, and these changes facilitate pathogen survival and various malignant behaviors (34).

miRNAs have recently been identified as critical regulators of lipid metabolic cycles, regulating the enzymes involved in lipid metabolism at the posttranscriptional level (35). This finding indicates that miRNAs are involved in the occurrence and development of various diseases by regulating lipid metabolism. The regulation of lipid metabolism by miR-33 has been intensively studied. Human miR-33 is located in the introns of the sterol regulatory element binding protein (SREBP) gene. Its mature form can be classified as miR-33a and miR-33b (36). miR-33 and its passenger strand (miR-33*) can target the key enzymes involved in cholesterol efflux, fatty acid metabolism, and insulin signaling, such as ATP binding cassette subfamily A member 1 (ABCA1), carnitine O-octanoyltransferase, and insulin receptor substrate 2 (37). A previous study reported that ABCA1 and ATP-binding cassette transporter G1 are targets through which miR-33a facilitates the SREBP-2-mediated regulation of cholesterol levels, thereby preventing the further removal of cholesterol from cells (38). Previous studies have reported that silencing miR-33 expression increases plasma high density lipoprotein (HDL) levels, reducing cholesterol flow to apolipoprotein A1 or neonatal HDL (39, 40). Many investigations have also indicated that the regulation of lipid metabolism by miR-33 is associated with many diseases. For example, several studies have shown that abnormal cholesterol metabolism is associated with neurodegenerative conditions, such as Alzheimer’s disease (41, 42) and age-related macular degeneration (43), and miR-33 is instrumental in these pathological processes.

Mtb interacts with the host through complex lipid components in its cell wall; these interactions regulate metabolism and immune responses, thus affecting the physiological processes of the host cell and Mtb itself. Furthermore, since host lipids are the main source of nutrition for Mtb, the host can affect the outcome of an infection by regulating lipid homeostasis (44). Previous studies have shown that human macrophages infected with Mtb are induced to form lipid droplets (45). Moreover, high expression of lipid sequestration- and metabolism-related genes is observed in human TB granulomas, suggesting that the development of TB is related to the dysregulation of host lipid metabolism (46). It has also been reported that Mtb can not only use host intracellular lipid droplets as nutrients but also respond to host immune mechanisms by controlling the lipid contents in its cell wall (46). After murine peritoneal macrophages and macrophages derived from a transformed human mononuclear cell line (THP-1) are infected with Mtb for 48 h, miR-33 and miR-33* expression is upregulated through nuclear factor NF-kappa-B (NF-κB)-dependent mechanisms. After their upregulation, miR-33 and miR-33* negatively regulate mitochondrial fatty acid oxidation and extend lipid storage in macrophages. Finally, Mtb uses these macrophage lipids as a source of nutrients for its growth and reproduction (25). Although the upregulation of miR-33 favors the intracellular survival of Mtb, the effect of miR-33 overexpression on Mtb survival is still unclear. Overexpression of miR-33 may be detrimental to lipid storage in macrophages or may activate other immune responses in the host, which are not beneficial to the survival of Mtb. The effect of miR-33 overexpression on Mtb survival needs further study.

### miRNAs modulate inflammatory responses

Inflammation is a defense response to stimuli and a basic physiological process that maintains homeostasis (46). When the body is subjected to physical damage, harmful stimuli (chemicals), pathogen invasion, tissue necrosis, or other harmful conditions that disrupt tissue homeostasis, the body initiates the inflammatory response (47). The typical inflammatory response involves several processes: the production of inflammatory inducers and the sensors that detect them, the production of inflammatory mediators, and the channeling of inflammatory mediators to target tissues (48). Moreover, it has been reported that complexes of soluble factors interact with cells during inflammation, which is a host response that accounts for tissue damage (49). These responses lead to the main manifestations of inflammation: redness, swelling, heat, pain, and dysfunction (50).

Toll-like receptors (TLRs), which are one group of pattern recognition receptors, bind to pathogen-associated molecular patterns and induce the activation of multiple proinflammatory factors through the NF-κB and mitogen-activated protein kinase-related signaling pathways, resulting in inflammatory responses and pathogen clearance. Tumor necrosis factor-alpha (TNF-α), IL-1, IL-6, and IL-8 are among the inflammatory factors that are produced after TLR activation, and these factors subsequently play major roles as inflammatory cytokines. Members of the IL-1 family are central mediators of innate immunity and inflammation; most IL-1 family members (IL-1α, IL-1β, IL-18, IL-33, IL-36α, IL-36β, and IL-36γ) have proinflammatory activities, whereas some (IL-37 and IL-38) have anti-inflammatory effects (51). Previous studies have reported that cytokines and receptors of the IL-1 family are potent factors that prime and amplify the immune response, affecting nearly all cells that are involved in the innate immune system (52, 53). IL-6 can be produced by various cells, including monocytes, macrophages, dendritic cells (DCs), T cells, and B cells (54). Specifically, IL-6 exerts several effects on both immune and nonimmune cells (55); it can drive B-cell precursors to become antibody-producing cells (56), facilitate the growth and differentiation of primitive bone marrow-derived cells and enhance the lysis function of natural killer (NK) cells (57). In contrast, TNF-α is another prominent inflammatory mediator of inflammatory responses (58) that can activate neutrophils and lymphocytes, increase the permeability of vascular endothelial cells, regulate metabolic activities of other tissues, and promote the synthesis/release of other cytokines (59). Consequently, the presence of many inflammatory cytokines makes the body’s immune response function properly.

When an organism is infected with a pathogen, the following occur: pathogen invasion, pathogen colonization of host tissue, immune response induction, pathogen clearance, or tissue damage. Inflammation is a link between innate and acquired immunity, helping the body to further eliminate pathogenic microorganisms and stimulating the initiation of acquired immune responses. Notably, intracellular bacteria multiply in host cells to escape attack by phagocytes, complement components, and antibodies. The common target cells of intracellular bacteria are epithelial cells, endothelial cells, hepatocytes, and macrophages (60). As a result, these bacteria, such as Mtb, *Listeria*, and *Mycobacterium leprae*, are difficult to eliminate from the host due to their intracellular life cycles. Subsequently, a structure called a granuloma forms in the infected area of the host when the host immune function is overwhelmed by the pathogen, resulting in chronic infection (61). Nevertheless, a few other pathogens survive and lie dormant in granulomas. If the granuloma ruptures, the pathogen can be reactivated and begin to proliferate (62). A previous study reported that when infected with Mtb, DCs, macrophages, and CD4^+^ T cells produce TNF-α and IL-12 in large quantities (63). Thus, NK cells enhance the ability of macrophages to phagocytose and kill pathogens by producing IFN-γ (64). The production of proinflammatory factors, such as IFN-γ, IL-12, and TNF, is essential for controlling Mtb infection (65). It was previously reported that miRNAs also play critical roles in controlling Mtb infection by regulating the inflammatory response and cytokine signal activation (66).

A summary of the miRNAs that modulate the inflammatory response during Mtb infection is shown in **Table 1**. Among the 24 miRNAs listed, 17 are upregulated and 7 are downregulated during Mtb infection. In macrophages, which are the main host cell of Mtb, eight miRNAs (miR-26a, miR-29a-3p, miR-32-5p, miR-125-5p, miR-132-3p, miR-155, miR-203, and miR-1178) (67–74) are upregulated and six miRNAs (miR-149, let-7 family, miR-20b, miR-27a, miR-18b, and miR-142-3p) (83–87, 89) are downregulated. However, in other types of immune cells, nine miRNAs (miR-21-5p, miR-99b, miR-144*, miR-30, miR-206, miR-140, miR-29b-1*, miR-124, and miR-223) (30, 75–82) are upregulated, and one miRNA (miR-378d) (88) is downregulated.

**Table 1:** microRNAs modulate the inflammatory response after Mtb infection.

microRNA	Cell type	Expression of micro-RNA	Up- or down-regulated micro-RNA regulates the inflammatory factors	Function of up- or down-regulated micro-RNA	Reference
miR-21-5p	APCs, BMDCs	Upregulated	Inhibits IL-12	Promotes Mtb survival	(30)
miR-26a	Macrophages	Upregulated	Inhibits IFN-γ	Promotes Mtb survival	(67)
miR-29a-3p	Macrophages	Upregulated	Inhibits IFN-γ and TNF-α	Promotes Mtb survival	(68)
miR-32-5p	Macrophages	Upregulated	Inhibits IL-1β, IL-6, and TNF-α	Promotes Mtb survival	(69)
miR-125-5p	Macrophages	Upregulated	Inhibits IFN-γ and TNF-α	Promotes Mtb survival	(70)
miR-132-3p	Macrophages	Upregulated	Inhibits IFN-γ	Promotes Mtb survival	(67)
miR-155	Macrophages	Upregulated	Promotes IFN-γ while inhibits IL-6 and IL-10	Unique dual action	(71, 72)
miR-203	Macrophages	Upregulated	Inhibits NF-κB, TNF-α, and IL-6	Promotes Mtb survival	(73)
miR-1178	Macrophages	Upregulated	Inhibits IFN-γ, IL-6, IL-1β, and TNF-α	Promotes Mtb survival	(74)
miR-99b	DCs, macrophages	Upregulated	Inhibits TNF-α, IL-6, IL-12, and IL-1β	Promotes Mtb survival	(75)
miR-144*	T cells	Upregulated	Inhibits TNF-α and IFN-γ	Promotes Mtb survival	(76)
miR-30	THP-1 cells	Upregulated	Inhibits TNF-α, IL-6, and IL-8	Promotes Mtb survival	(77)
miR-206	THP-1 cells	Upregulated	Promotes IL-1β, IL-6, IFN-γ, and TNF-α	Inhibits Mtb survival	(78)
miR-140	PBMCs, THP-1 cells	Upregulated	Inhibits IFN-γ, IL-6, IL-1β, and TNF-α	Promotes Mtb survival	(79)
miR-29b-1*	NKs, CD4^+^T cells	Upregulated	Inhibits IFN-γ	Promotes Mtb survival	(80)
miR-124	Peripheral leukocytes, AMs	Upregulated	Inhibits IL-6 and TNF-α	Promotes Mtb survival	(81)
miR-223	Neutrophil, myeloid cells	Upregulated	Inhibits IL-6	Promotes Mtb survival	(82)
miR-149	Macrophages	Downregulated	Promotes TNF-α, IL-1β, and IL-6	Inhibits Mtb survival	(83)
let-7 family	Macrophages	Downregulated	Inhibits TNF and IL-1β	Promotes Mtb survival	(84)
miR-20b	Macrophages	Downregulated	Promotes IL-18 and IL-1β	Inhibits Mtb survival	(85)
miR-27a	Macrophages	Downregulated	Promotes IFN-γ, IL-β, IL-6, and TNF-α	Inhibits Mtb survival	(86)
miR-18b	Macrophages	Downregulated	Promotes IL-6	Inhibits Mtb survival	(87)
miR-378d	THP-1 cells	Downregulated	Promotes IL-1β, IL-6, and TNF-α.	Inhibits Mtb survival	(88)
miR-142-3p	Macrophages	Downregulated	Promotes TNF-α and IL-6	Inhibits Mtb survival	(89)

*represents miRNAs’ passenger strand

A previous study showed that the miR-155-dependent downregulation of Src homologous 2-inositol phosphatase-1 (SHIP-1) could play a role in the survival of Mtb in infected mouse macrophages (24 h postinfection). As a direct target of miR-155, SHIP1 downregulation promotes the activation of serine/threonine kinase AKT, which is beneficial for Mtb survival (90). In adaptive immunity, miR-155 enhances IFN-γ production by human CD8+ and CD4+ T cells by targeting cytokine signaling-1 (91). However, miR-155 plays a dual regulatory role. Although miR-155 maintains bacterial survival in the early stage of macrophage infection, it promotes IFN-γ production by T cells to control Mtb infection in later stages (72). It may be that miR-155 plays different roles in innate immunity and adaptive immunity. miR-21 regulates not only host glycolysis but also inflammatory responses. After infection of RAW264.7 and THP-1 cells with Mtb for 24 h, the expression of miR-21-5p increases dramatically. miR-21-5p directly targets Bcl-2 and TLR4 in Mtb-infected macrophages to reduce the secretion of the inflammatory cytokines TNF-α, IL-1β, and IL-6, thereby allowing Mtb to evade the host immune response (92). miR-125b has been reported to directly target the 3’-UTR of κB-RAS2, an inhibitor of NF-κB signaling, increasing its stability and reducing inflammatory responses in primary human macrophages (91). Accordingly, in peripheral blood mononuclear cells of TB patients, miR−125b plays a vital role in the development and progression of TB by reducing the IFN-γ, IL-6, TNF-α, and NF-κB levels by inhibiting the Raf1 proto−oncogene serine/threonine protein kinase (93). As a major regulator of the cellular oxidative stress response, miR-144 directly targets nuclear factor erythroid 2-related factor 2 to modulate the oxidative stress response (94). The levels of miR-144*, which is the passenger strand of miR-144, are significantly elevated in the blood of TB patients, and this molecule regulates cytokine production by T cells (95). A previous study reported that miR-144* might regulate anti-TB immune responses by blocking the production of TNF-α/IFN-γ and inhibiting the proliferation of human T cells (76). However, no study has elucidated the specific mechanism by which miR-144* inhibits T-cell proliferation. The effect of miR-144* on T cell proliferation requires further study. In addition, miR-223, which is a small noncoding RNA, has been shown to be upregulated in the lung parenchyma and blood of patients with TB (82). In addition, studies have suggested that IL-6, chemokine ligand 3, and chemokine ligand 2 are novel targets of miR-223 (82, 96). Another previous study identified miR-29 as a central inhibitor of IFN-γ (68). As previously reported, the expression of miR-29 and IFN-γ is negatively correlated with Mtb infection, and miR-29 is downregulated in IFN-γ-secreting T cells and NK cells (97, 98). Moreover, promoting miR-29 expression increases susceptibility to mycobacterium infection (80). miR-29 has been identified as a regulator that suppresses immune responses to intracellular pathogens. Although miR-27a is expressed at low levels in Mtb-infected THP-1 macrophages (99), a previous study reported that miR-27a reduces the levels of IFN-γ, IL-β, IL-6, and TNF-α in macrophages by targeting IL-1 receptor-activated kinase 4 (an important kinase in the immune response), inhibiting the immune response, and enhancing the survival rate of intracellular Mtb (86). Similarly, miR-18b is downregulated in Mtb-infected human and murine macrophage cell lines. Recent studies have confirmed that low expression of miR-18b promotes the expression of hypoxia-inducible factor 1α, induces the production of proinflammatory cytokines and reduces the viability of bacteria in host cells (87). The levels of miR-20b, a member of the miR-17 family, are decreased in the serum and macrophages of patients with TB (100). A previous study reported that the activation of the NLR family pyrin domain containing 3 (NLRP3) facilitates the maturation of IL-1β and IL-18, eventually enhancing innate immune defenses (101). Furthermore, downregulation of miR-20b and upregulation of NLRP3 are observed in the macrophages of TB patients. In a TB mouse model, miR-20b was shown to directly bind to the 3’-UTR of NLRP3 and negatively regulate its expression. In summary, the downregulation of miR-20b increases the expression of NLRP3 and activates the NLRP3/caspase-1/IL-1β pathway to inhibit Mtb survival in macrophages (85). Previous studies have also indicated that miR-142-3p can target and inhibit the expression of IL-6 (102). Additionally, although miR-142-3p is downregulated in the peripheral CD4^+^ T cells and macrophages of patients with TB (89, 103), miR-142-3p expression is negatively correlated with the production of the proinflammatory mediators IL-6, NF-κB, and TNF-α. Therefore, decreasing miR-142-3p expression could delay the survival of Mtb in macrophages (89, 104). After infection of THP-1 and RAW264.7 macrophages with Mtb for 24 h, the downregulation of miR-378d causes the increase in Rab10 expression, which in turn leads to the increased expression of TLR4 on the cell surface and activation of the NF-κB, interferon regulatory factor 3, and MAPK signaling pathways (88, 105). As a result, a decrease in miR-378d expression can promote the production of the cytokines IL-1β, IL-6, and TNF-α, which is conducive to the clearance of intracellular Mtb.

## Regulation of autophagy by microRNAs

### Targeting ATG affects autophagy

Autophagy is a biological process that degrades intracytoplasmic macromolecules and organelles in capsular vesicles (106). During autophagy, part or all of the cytoplasm, including its organelles, are enclosed in double-membrane vesicles, forming autophagic vacuoles or autophagosomes. Soon after these autophagosomes are formed, they become monolayers and then combine with lysosomes to form autophagolysosomes (107). In autophagolysosomes, substances are decomposed into amino acids and nucleotides by various enzymes, and these amino acids and nucleotides can enter the tricarboxylic acid cycle to generate small molecules and energy (108). Additionally, the process of autophagy mainly involves the following stages: nucleation, elongation, formation and maturation of autophagosomes followed by fusion of autophagosomes and lysosomes. All these stages involve many genes, such as Beclin 1, AMP-activated protein kinase (AMPK), mammalian target of rapamycin complex 1 and autophagy-related genes (ATG) (109–111). Dozens of ATG and their homologs have been identified. A previous study reported that the whole process of autophagy is regulated by different ATGs (109).

Autophagy targets bacteria in the cytoplasm or vacuoles, and this selective type of autophagy can be called xenophagy (112). A previous study reported that microtubule-associated protein light chain 3 (LC3)-modified autophagosomes form around target bacteria, and the pathogens are degraded through this LC3-associated phagocytotic process by promoting lysosome fusion with phagosomes (113). Moreover, increasing evidence suggests that autophagy can eliminate pathogens, but these pathogens can use various strategies to avoid being killed and to escape from phagosomes. Hence, pathogens block phagosome maturation, allowing their long-term survival in phagosomes (114). For example, *Shigella foestri* can competitively bind to the cell surface virulence protein IcsA through the invasion protein IcsB and block the binding of IcsA and ATG5 to avoid autophagic degradation (115). *Listeria monocytogenes* can also escape phagosomes *via* a toxin that forms pores in the phagosome membrane, enter the cytoplasm, and use the cell surface protein actin assembly inducing protein ActA to recruit host actin to the bacterial surface to prevent its recognition by autophagic machinery (116). Similarly, it has been demonstrated that Mtb has developed several strategies to evade autophagy. Among these strategies, Mtb can survive in cells by regulating miRNA expression profiles to avoid immune attack (117).

As previously reported, the microtubule-associated protein LC3-I binds to phosphatidylethanolamine *via* a ubiquitin-like reaction that requires Atg7 and Atg3 (E1- and E2-like enzymes) (118). Mtb also reduces the Atg3 protein content through miR-155, negatively regulating autophagy (119). Moreover, the silencing of miR-155 during Mtb infection rescues autophagosome formation (119). Another previous study demonstrated that miR-155 is highly expressed in Mtb-infected mouse macrophages and enhances autophagy by targeting Rheb to inhibit Mtb survival (120). In addition, among the ATGs, Atg4 is a protease that is involved in converting LC3-I to LC3-II (121). A previous study also suggested that miR-129-3p is a repressor that facilitates the survival of Mtb in macrophages by targeting Atg4b-mediated autophagy (122). Similarly, miR-144-3p represses Atg4a expression by targeting its 3’-UTR, hindering the activation of autophagy (123). Atg7 has been reported to be involved in autophagosome formation and vesicle progression (124), whereas Atg16L1 controls the extension of nascent autophagosome membranes (125), indicating that Atg7 and Atg16L1 play essential roles in autophagy. As a member of the miR-17 family (126), there is increasing evidence that miR-106a regulates autophagy by targeting unc-51 like autophagy activating kinase 1 (ULK1), Atg7, and Atg16L1 (127, 128). A related study also indicated that miR-106a acts as a negative regulator of autophagy during Mtb infections, downregulating the expression of autophagy proteins by targeting ULK1 Atg7 and Atg16L1, thus inhibiting the autophagic process in macrophages (129). miR-20a can also target Atg7 and Atg16L1 to regulate autophagy and promote Mtb survival (130). Atg5 is a key protein involved in the elongation of phagocytic membranes in autophagic vesicles, and it forms a constitutive complex with Atg12 (131). Atg12-Atg5 then further binds to Atg16L to form an Atg12-Atg5-Atg16L complex, which is located on the outer membrane of the autophagosome (132). However, miR-1958 reduces the expression of Atg5 by interacting with Atg5’s 3’-UTR, inhibiting autophagy and promoting the survival of intracellular Mtb (133).

### miRNAs affect autophagy by regulating other factors

**Figure 1:**
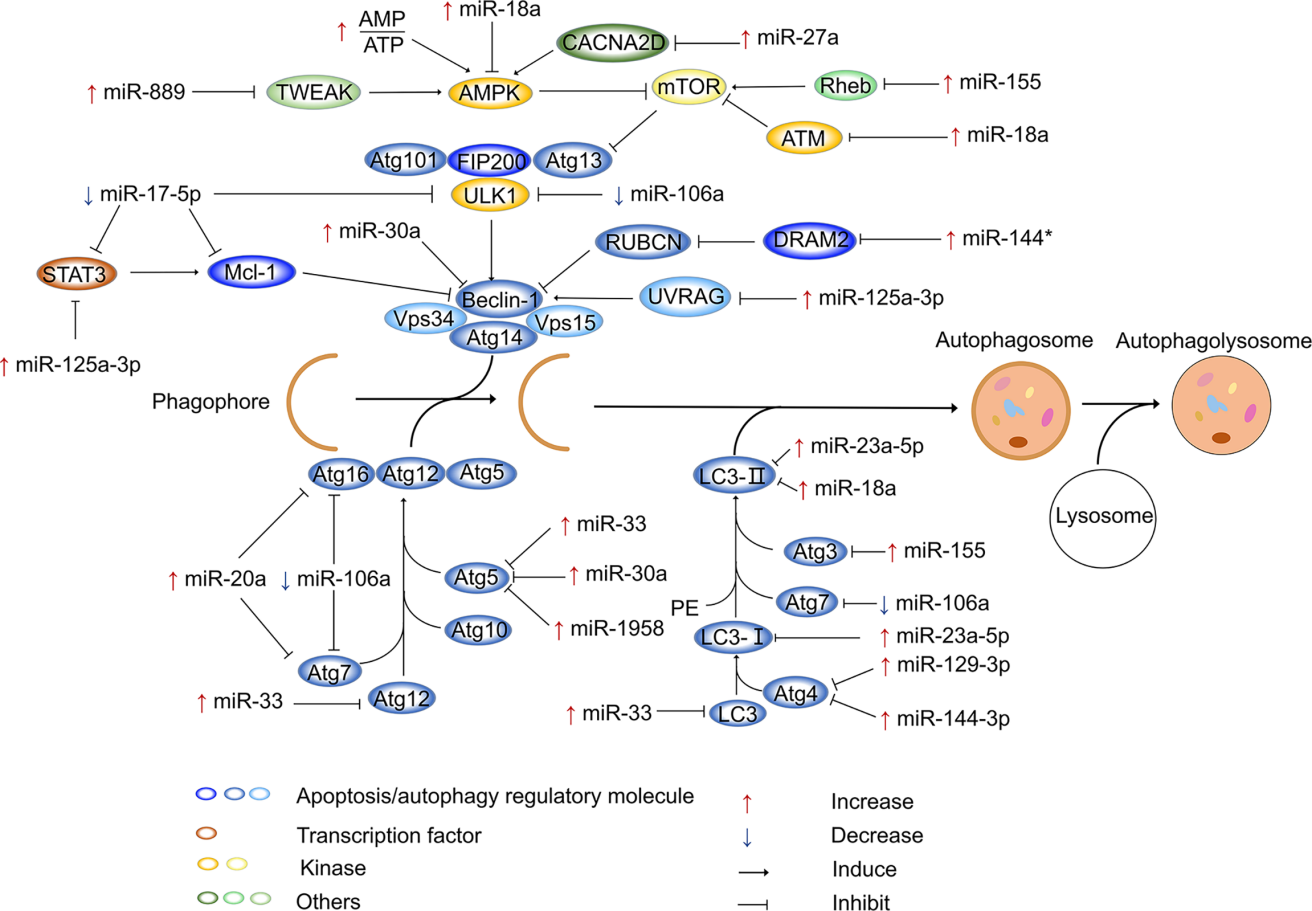
miRNAs regulate autophagy after Mtb infection. In the process of autophagy, membrane vesicles encapsulate cytoplasm and organelles to form autophagosomes, which then fuse with lysosomes to form autophagolysosomes. After Mtb infection, miRNAs inhibit or activate autophagy process by modulating ATG genes and other autophagy factors. In this process, various miRNAs are mainly involved in the formation of autophagosomes. The host can defend against the invasion of Mtb by miRNAs. In addition, Mtb also develops strategies to combat autophagy by modulating miRNA expression. “Increase” represents the upregulation of miRNAs, “Decrease” represents the downregulation of miRNAs. “Induce” represents miRNAs promote gene expression, “Inhibit” represents miRNAs suppress gene expression , *represents miRNAs’ passenger strand.

In addition to targeting ATG to regulate intracellular autophagy, miRNAs can regulate autophagy by targeting other components. For example, intracellular Ca^2+^ signaling regulates many basic cellular processes (134), and increasing evidence suggests that Ca^2+^ is a secondary messenger that regulates intracellular autophagy (135, 136). It has also been reported that calcium channels mediate the influx of calcium ions into cells upon membrane polarization. Elevated miR-27a expression has been observed in Mtb-infected cells, infected animals, and patients with active TB. Moreover, miR-27a directly targets calcium voltage-gated channel auxiliary subunit alpha2delta 3 (a Ca2+ transporter in the endoplasmic reticulum), inhibits the endoplasmic reticulum (ER) Ca^2+^ signaling pathway to reduce autophagy, and facilitates the intracellular survival of Mtb (137). Evidence has also suggested that the 3p and 5p arms of miRNAs perform the same or opposite functions in regulating gene expression (138, 139); this phenomenon was also observed for miR-30a. Specifically, miR-30a-3p provides a survival advantage for invading Mtb by inhibiting the maturation of autophagosomes and the fusion of mature autophagosomes with lysosomes (140). However, the activation of miR-30a-5p enhances autophagy, ultimately decreasing the growth of intracellular mycobacteria (141). It has been demonstrated that Mtb infection leads to downregulation of miR-17, which is accompanied by the upregulation of its target myeloid cell leukemia sequence 1 (Mcl-1) and signal transducer and activator of transcription 3 (STAT3, a transcriptional activator of Mcl-1) (142). Overexpression of miR-17 decreases the phosphorylation of protein kinase C-δ (an activator of STAT3) and the expression of Mcl-1 and STAT3. This suggests that during Mtb infection, downregulation of miR-17 inhibits autophagy through the miR-17–PKC-δ–STAT3–Mcl-1 pathway (143). A previous study demonstrated that TNF superfamily member 12 (TWEAK) enhance the expression of ATG in myotubes (144), suggesting that TWEAK may be involved in the regulation of autophagy. TWEAK is upregulated by mycobacterium components (Ag85A and Ag85B), and upregulated TWEAK induces phagosome maturation and promotes autophagy, ultimately decreasing intracellular mycobacterium survival (144). Increased miR-889 expression is observed in TB patients, and miR-889 inhibits autophagy to maintain the survival of Mtb *via* the posttranscriptional inhibition of TWEAK expression (145). Previous studies have also reported that UV radiation resistance associated gene (UVRAG) is involved in autophagy maturation and transport of endocytic vesicles to accelerate endocytic degradation (146, 147). UVRAG is crucial in the induction of autophagy, and it is the direct target of miR-125a-3p. After infection of mouse macrophages (RAW264.7 cells and BMDMs) with Mtb for 24 h, the increased expression of miR-125a-3p inhibits autophagy activation and antimicrobial effects against Mtb by targeting UVRAG (148). miR-125a-5p enhances autophagy by targeting the inhibition of STAT3 expression and blocks the intracellular survival of Mtb (149). In mouse macrophages, Mtb infection increases the expression of miR-23a-5p in a time- and dose-dependent manner (150). It has been reported that miR-23a-5p interacts with the 3’-UTR of Toll-like receptor 2 to inhibit its expression, impairing the TLR2/myeloid differentiation primary response gene 88 (MyD88)/NF-κB pathway and promoting the survival of Mtb (150). miR-18a belongs to the miR-17 family (151), and its expression gradually increases within 24 h after infection of RAW264.7 cells with Mtb. miR-18a directly targets and downregulates ataxia telangiectasia mutated (ATM) to inhibit autophagy and promote mycobacterial survival in macrophages. Furthermore, inhibition of miR-18a upregulates p-AMPK expression, which can be reversed by downregulating ATM. Therefore, the increased expression of miR-18a inhibits autophagy through the ATM-AMPK pathway, ultimately promoting intracellular Mtb survival (152). Similarly, DNA damage regulated autophagy modulator 2 (DRAM2) is a transmembrane lysosomal protein that is associated with autophagy processes (153), and it can interact with UVRAG to induce autophagy. miR-144* is expressed at notably high levels in Mtb-infected cells and interacts with the 3’-UTR of DRAM2 to reduce DRAM2 expression and autophagosome formation. As a result, miR-144* can decrease the antimicrobial response to Mtb by targeting DRAM2 (154). A previous study proved that miR-125b-5p can also target DRAM2 to inhibit antimicrobial responses in macrophages (155). **Figure 1** shows the miRNAs involved in the regulation of autophagy after Mtb infection.

## Regulation of apoptosis by miRNAs

### Disease- and pathogen-mediated apoptosis of host cells

Apoptosis involves the activation, expression, and regulation of a series of genes. Apoptosis includes four stages: reception of apoptotic signals, interaction between apoptosis-regulating molecules, activation of proteolytic enzymes, and continuous reaction (156). Many studies have proven that apoptosis is induced by specific signals and that multiple genes coregulate apoptosis (157). For instance, the caspase gene family, p53 gene, Bcl-2 gene family, cellular myelocytomatosis viral oncogene, Fas cell-surface death receptor (Fas), and Fas ligand can all trigger apoptosis. Apoptosis is a physiological mechanism that maintains homeostasis. Some pathogenic factors inhibit or enhance apoptosis by targeting apoptosis-related genes, disrupting cell homeostasis and eventually causing various diseases (156). However, more apoptosis is not necessarily better. Excessive apoptosis exacerbates the outcomes of many diseases, such as neurodegenerative diseases, AIDS, and cardiovascular diseases (158). Insufficient apoptosis also results in disease. From the perspective of apoptosis, the pathogenesis of autoimmune diseases is caused by insufficient apoptosis and ineffective clearance of autoimmune T cells (159).

Additionally, apoptosis is crucial for the elimination of infected cells from the host. Activation of apoptosis can effectively remove infected cells and terminate infection. However, the induction of apoptosis does not always protect host cells from microbial infection. Viruses and bacteria can exploit the host’s apoptotic machinery to reduce the number of cells that are needed for an immune response, allowing intracellular pathogens to escape clearance mechanisms and survive (160). There is a close relationship between viral infection and apoptosis (161). Viruses can cause tissue damage by increasing apoptosis rates (162, 163). There are also some viruses (such as poxviruses, herpesviruses, and adenoviruses) whose genomes encode antiapoptotic proteins that facilitate the completion of the viral replication cycle before apoptosis (164, 165), thereby ensuring viral replication and reproduction (166). In 1992, scientists discovered that bacteria could cause the apoptosis of infected host cells, so research on apoptosis also began to involve the field of bacterial infection (167). Similar to viral infections, bacterial infections promote or inhibit apoptosis. Different bacteria can affect the regulation of apoptosis. A single type of bacteria with different degrees of virulence may also differently affect apoptosis.

Apoptosis has long been recognized to be an effective defense against the spread of mycobacterial infection (168). Mtb species with different degrees of virulence exert opposite effects on the apoptosis of macrophages. Attenuated mycobacteria induce apoptosis, and the growth of Mtb in macrophages is reduced, but Mtb is latent in macrophages (169, 170). Virulent Mtb blocks macrophage apoptosis, thereby maintaining its replicative niche and eventually causing host cell necroptosis to facilitate its escape and spread (171). Mtb utilizes multiple mechanisms to regulate host apoptosis. For example, the type VII secretion system ESX-1 secretion-associated protein EspC, a substrate protein secreted by Mtb, is thought to induce ER stress-mediated apoptosis (172). The type VII secretion system ESX-1 transcriptional regulator EspR is a DNA binding protein of Mtb that inhibits macrophage apoptosis through MyD88/TLR, providing opportunities for mycobacterial survival (173). The Mtb virulence factor phosphotyrosine protein phosphatase also promotes the intracellular survival of Mtb by inhibiting apoptosis (174). However, some proteins that are secreted by Mtb induce apoptosis (175–177).

### miRNAs regulate apoptosis

**Figure 2:**
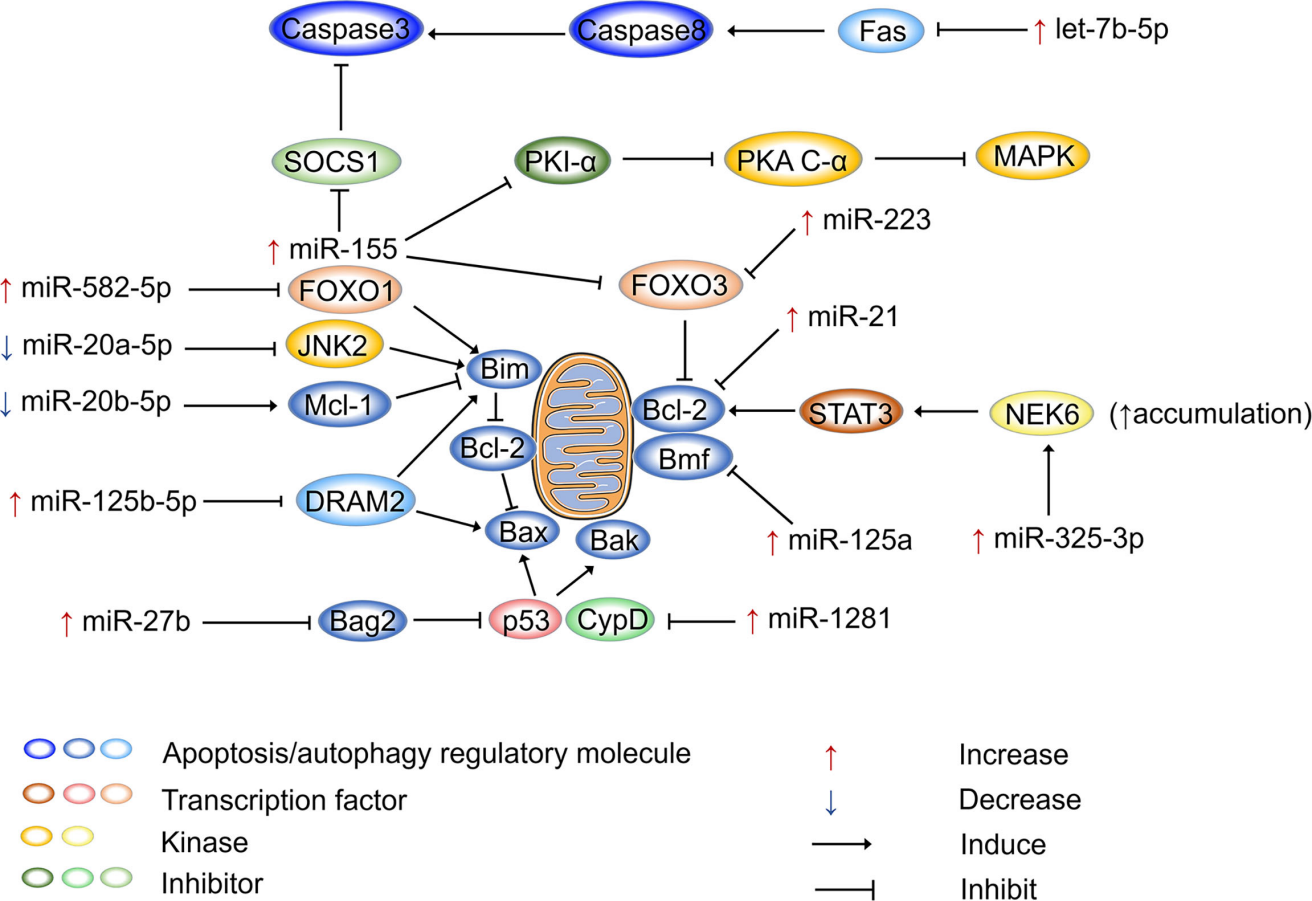
miRNAs regulate apoptosis after Mtb infection. miRNAs participate in apoptosis *via* apoptosis regulatory molecules after Mtb infection, and miRNAs are mainly involved in mitochondria-led apoptosis. Bcl-2 family proteins are the major regulators that control the release of mitochondria-associated apoptotic factors. Bax, Bim, and Bak are pro-apoptotic proteins that activate apoptotic pathways, while Bcl-2 plays an anti-apoptotic role. Mtb can regulate the mitochondria-led apoptosis of host cells by selectively regulating miRNA expression, thereby ensuring its intracellular replication and proliferation. “Increase” represents the upregulation of miRNAs, “Decrease” represents the downregulation of miRNAs. “Induce” represents miRNAs promote gene expression, “Inhibit” represents miRNAs suppress gene expression.

**Figure 2** shows how miRNAs regulate apoptosis in host cells after Mtb infection. miR-155 is not only an inflammatory regulator that performs dual functions but also plays dual regulatory roles in apoptosis. On the one hand, miR-155 promotes apoptosis to release Mtb antigens and activate T-cell immune function. On the other hand, it defends against apoptosis, allowing pathogens to escape and spread (178). miR-155 is upregulated in RAW264.7 macrophages after 12 h of *M. bovis* BCG infection. Furthermore, it has been shown that miR-155 enhances cAMP dependent protein kinase (PKA) signaling pathway activation by directly targeting protein kinase inhibitor alpha, which is a negative regulator of PKA signaling in macrophages. This process provides the main signal that drives macrophage apoptosis, resulting in loss of macrophage viability and favoring Mtb proliferation (179). Another study pointed out that miR-155 can inhibit apoptosis. miR-155 is upregulated in peripheral blood mononuclear cells (PBMCs) of patients with active TB, and it binds to the 3’-UTR of Forkhead box O3 (FOXO3) to inhibit its expression. It has been reported that the numbers of PBMCs in patients with active TB increase as a result of the inhibition of apoptosis by miR-155 (180). Similarly, miR-223 is upregulated in the monocyte-derived macrophages (MDMs) from patients with active TB and in infected THP-1 cells (181). miR-223 can also target FOXO3 to inhibit apoptosis (182). miR-20a-5p and miR-20b-5p are two highly homologous miRNAs, both belonging to the miR-17 family (183), but they perform different functions in regulating the apoptosis of Mtb-infected macrophages. The expression of miR-20a-5p is reduced in infected THP-1 macrophages, which is followed by c-Jun N-terminal kinase 2 (JNK2) and Bim activation. Mechanistically, miR-20a-5p directly targets JNK2 to regulate Bim expression, promoting apoptosis and Mtb clearance (184). Interestingly, miR-20b-5p downregulation causes effects that are diametrically opposed to those of miR-20a-5p. miR-20b-5p targets and negatively regulates Mcl-1, which increases cell viability and attenuates apoptosis in Mtb-infected macrophages (100). It has been demonstrated that miR-125b-5p is highly expressed in Mtb-infected macrophages and monocytes from TB patients. miR-125b-5p can target DRAM2 to decrease the expression of the apoptotic genes Bax and Bim, thereby inhibiting apoptosis (155). miR-21 acts as an apoptosis repressor in various tumor cells (185), and its antiapoptotic function is performed by upregulating the antiapoptotic factor Bcl-2 (186, 187). miR-21 also increases Bcl-2 expression in mouse monocyte macrophages (J774 macrophages) treated with the Mtb-derived protein Mpt64 (188). In BCG-infected mouse bone marrow-derived dendritic cells, miR-21 expression is increased, which inhibits Bcl-2, resulting in increased apoptosis (30, 92). miR-1281 can target and inhibit cyclophilin D, thereby protecting Mtb-infected human macrophages from programmed necrosis and apoptosis (189). The ligand of numb protein X 1 is an E3 ubiquitin ligase of NIMA related kinase 6 (NEK6) and a direct target of miR-325-3p. In Mtb-infected host cells, miR-325-3p is upregulated and blocks NEK6 degradation. Accumulation of NEK6 activates the antiapoptotic signal transducer and activator of transcription 3 signaling pathway, thereby promoting intracellular survival and the immune escape of Mtb (190). FOXO1 is considered to be a tumor suppressor and plays a proapoptotic role in various cells (191). miR-582-5p is abundantly expressed in the monocytes of patients with active TB and suppresses monocyte apoptosis by downregulating FOXO1 (192).

## miRNAs function as biomarkers

### miRNA expression after Mtb infection

Biomarkers refer to biochemical molecules that indicate changes in a system, organ, tissue, cell, and subcellular structure or function. Biomarkers can help diagnose disease, determine disease staging, and evaluate the safety and efficacy of new drugs or treatments in target populations. Biomarkers not only include biological macromolecules, such as proteins and nucleic acids, but also include proteomes and metabolomes (193). They can be obtained from blood, urine, saliva, cancer cells, and cancer tissue samples. According to their functions, biomarkers can be classified into six categories: biomarkers for diagnosis, prognosis, prediction, efficacy, safety, and monitoring.

**Figure 3:**
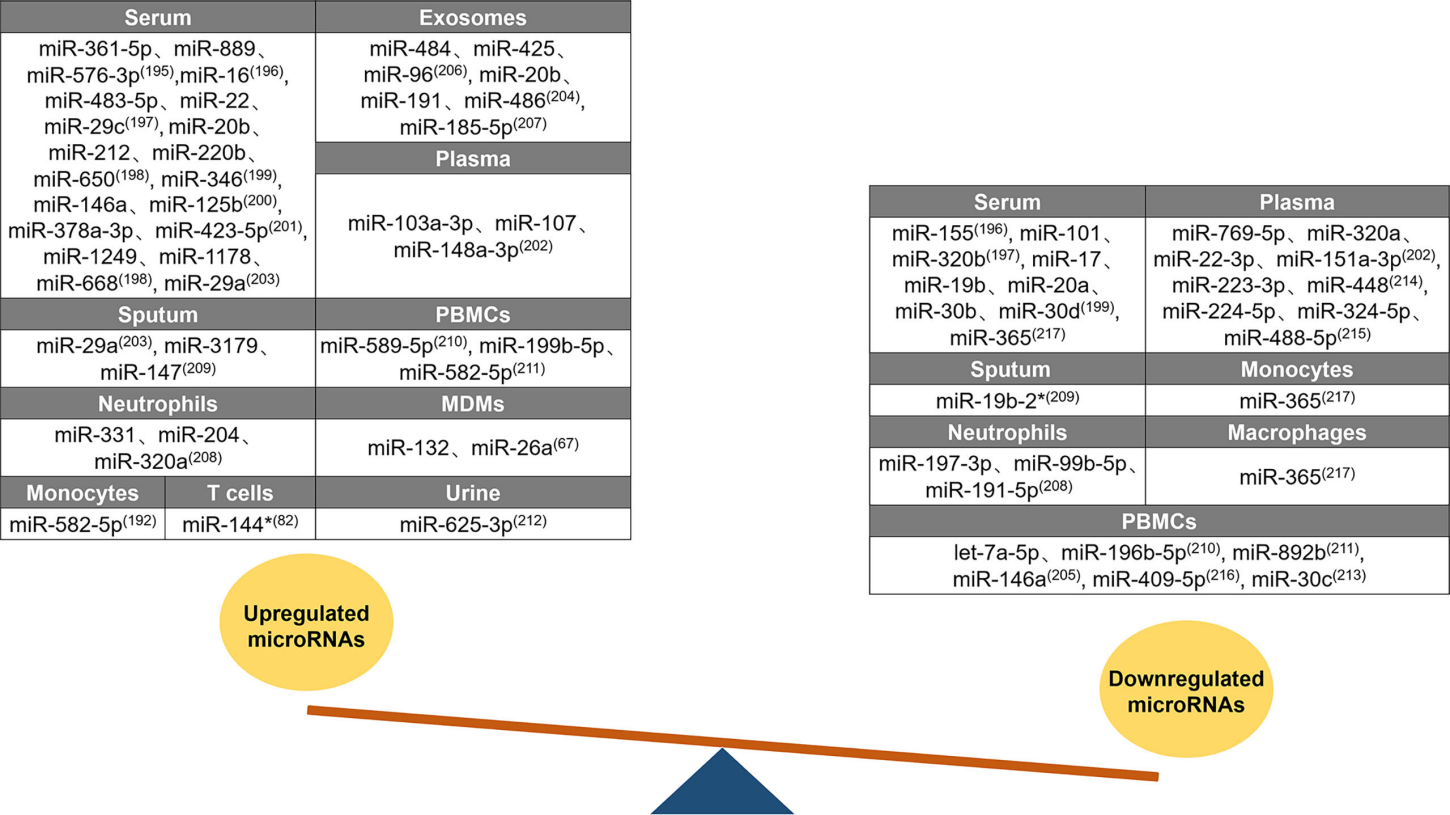
Differential expression of miRNAs after Mtb infection. Differential expression of miRNAs between tuberculosis patients and healthy individuals. Forty-three upregulated miRNAs are counted in serum, exosomes, plasma, sputum, PBMCs, neutrophils, MDMs, monocytes, T cells, and urine. Twenty-eight downregulated miRNAs are counted in serum, plasma, sputum, monocytes, neutrophils, macrophages, and PBMCs. Similar expression profiles of miRNAs (miR-29, miR-582-5p, miR-20b, miR-320, miR-19b, miR-30, and miR-365) are counted in multiple samples, while five miRNAs (miR-22, miR-20, miR-146a, miR-191, and miR-320) are with distinctive expression trends in different samples.

Changes in host miRNA expression have diagnostic potential in TB (194). A summary of the differences in miRNA expression between patients with active TB and healthy individuals is shown in **Figure 3**. Forty-three upregulated miRNAs and twenty-eight downregulated miRNAs were identified in different tissues of patients with active TB. Twenty upregulated miRNAs were identified in the serum of patients with active TB, of which 15 (miR-361-5p, miR-889, miR-576-3p, miR-16, miR-483-5p, miR-212, miR-220b, miR-650, miR-346, miR-125b, miR-378a-3p, miR-423-5p, miR-1249, miR-1178, and miR-668) are only upregulated in the serum (195–201). In patients with active TB, miR-22 expression is increased in the serum, but miR-22-3p is decreased in the plasma (197, 202). The miR-29 family members miR-29a and miR-29c are both upregulated in the serum and sputum of patients with active TB (197, 203). High levels of miR-20b are found in the serum and exosomes of patients, but opposite miR-20a expression patterns are found in serum (198, 204). Similarly, miR-146a expression in various tissue samples also follows diametrically opposing trends, and it increases in the serum and decreases in PBMCs (200, 205). Three miRNAs (miR-103a-3p, miR-107, and miR-148a-3p) are elevated only in the plasma of patients with active TB (202). The exosomal levels of miR-484, miR-425, miR-96, miR-486, and miR-185-5p increase in patients with active TB (204, 206, 207). For patients with active TB, the content of miR-191 is increased in exosomes and reduced in neutrophils (204, 208). Compared to unaffected healthy controls, miR-3179 and miR-147 levels in sputum (209), miR-589-5p and miR-199b-5p levels in PBMCs (210, 211), miR-331 and miR-204 levels in neutrophils (208), and miR-132 and miR-26a levels in MDMs all increased (67). miR-582-5p is increased in monocytes and PBMCs from patients with active TB (192, 211). miR-320 has unique expression patterns in patients; miR-320a is upregulated in neutrophils but downregulated in plasma, and miR-320b is downregulated in serum (197, 202, 208). miR-144* and miR-625-3p expression levels are increased in the T cells and urine of patients with active TB, respectively (76, 212). Three miRNAs (miR-155, miR-101, and miR-17) were only reduced in patient serum (196, 197, 199). The miR-30 family includes miRNAs that are downregulated in patients with active TB. For example, the expression of miR-30b and miR-30d in the serum and the expression of miR-30c in PBMCs are decreased (199, 213). In patients with active TB, seven miRNAs (miR-769-5p, miR-151a-3p, miR-223-3p, miR-448, miR-224-5p, miR-324-5p, and miR-488-5p) exhibited decreased levels only in the plasma (202, 214, 215), four miRNAs (let-7a-5p, miR-196b-5p, miR-892b, and miR-409-5p) exhibited decreased levels only in PBMCs (210, 211, 216), and two miRNAs (miR-197-3p and miR-99b-5p) exhibited decreased levels only in neutrophils (208). miR-365 is downregulated in the serum, monocytes, and macrophages of patients with active TB (217). Among the miRNAs summarized above, there are five miRNAs (miR-22, miR-20, miR-146a, miR-191, and miR-320) with distinctive expression patterns in different tissue samples, and these miRNAs may be useful as biomarkers to distinguish patients with active TB from healthy individuals. miRNAs can be used as markers for the early diagnosis of TB.

miRNAs can be used not only to identify TB patients but also to monitor treatment effects, drug resistance, and Mtb virulence in TB patients (**Table 2**). After anti-TB treatment, the expression of most miRNAs continued to be downregulated and gradually returned to the levels observed in uninfected healthy controls. The expression of seven miRNAs (miR-29a-3p, miR-155-5p, miR-361-5p, miR-99b, miR-29a, miR-146a, and miR-26) decreased in the plasma of treated patients (218, 219). Five downregulated miRNAs (miR-326, miR-346, miR-21-5p, miR-92a-3p, and miR-148b-3p) were identified in serum (199, 220, 221). Studies have shown that the miR-199b-3p, miR-199a-3p, and miR-16-5p levels in whole blood and the miR-424 levels in PBMCs are significantly decreased (222, 223). Increased miR-125a-5p levels were observed in TB patients who were treated for 2 months (221). As a unique miRNA in patients after treatment, miR-125a-5p expression can be combined with the greatly attenuated expression of other miRNAs to distinguish TB patients before and after treatment. Moreover, measuring miRNA expression can also Ibe used to determine the prognosis of TB patients.

**Table 2:** microRNAs as biomarkers in TB.

Content of comparison	Sample	Upregulated micro-RNAs	Downregulated microRNAs	Reference
Treated versus untreated	Plasma		miR-29a-3p、;miR-155-5p miR-361-5p	(218)
			miR-99b、;miR-29a miR-146a、;miR-26	(219)
	Serum		miR-326	(220)
			miR-346	(199)
		miR-125a-5p	miR-21-5p、;miR-92a-3p miR-148b-3p	(221)
	PBMCs		miR-424	(222)
	Blood		miR-199b-3p、;miR-199a-3p miR-16-5p	(223)
Drug-resistant versus pan-susceptible TB patients	Plasma		miR-320a	(202)
	Serum	miR-4433b-5p、;miR-424-5p		(224)
	Exosomes	let7e-5p	miR-197-3p、;miR-223-3p	(225)
Virulent versus avirulent	Macrophages		miR-145	(226)
	MDMs	miR-125b		(70)
	THP-1 cells	miR-4668-5p、;miR-30e miR-1275、;miR-30a miR-3178	miR-4484	(227)

Antibiotics are an effective means of treating TB. However, due to the abuse of antibiotics or the insufficient course of treatment used by patients, common TB has developed drug resistance. For example, Mtb has acquired streptomycin resistance mechanisms (228). Drug resistance can be divided into monodrug, multidrug, and extensive multidrug resistance (229). The emergence of drug-resistant bacteria has greatly affected the treatment of TB. Therefore, the accurate diagnosis of drug-resistant TB and effective treatment are key factors in blocking the spread of drug-resistant Mtb. This article summarizes seven miRNAs that exhibit differential expression in patients with common and drug-resistant TB. The miR-4433b-5p and miR-424-5p levels in serum and the let7e-5p levels in exosomes are decreased in pan-susceptible TB patients compared to drug-resistant TB patients (224, 225). The miR-320a levels in the plasma and the contents of miR-197-3p and miR-223-3p in exosomes of drug-resistant patients are reduced (202, 225). miRNAs can be used to identify drug-resistant patients in order to select appropriate treatment regimens as soon as possible and design chemotherapy regimens according to the patient’s medication history, the prevalence of drug-resistant strains, and the available drugs. However, the use of miRNAs to diagnose drug resistance is limited, and changes in miRNA expression patterns caused by different drug-resistant strains and differences among drug-resistant patients also vary. The identification of broad-spectrum miRNAs that are applicable to all drug-resistant strains will help in the diagnosis of drug-resistant individuals, and the identification of specific miRNAs that can be used as biomarkers of different drug-resistant strains will be helpful for developing targeted therapies.

Distinctive miRNA expression patterns are observed after infection with virulent (Mtb H37Rv), avirulent Mtb (Mtb H37Ra), and nonvirulent vaccine strains (M. bovis BCG). miR-145 expression in macrophages and miR-4484 expression in THP-1 cells is decreased after infection with the virulent strain (226, 227). Host cells upregulate six miRNAs (miR-125b, miR-4668-5p, miR-30e, miR-1275, miR-30a, and miR-3178) to inhibit the effects of the virulent strain (70, 227). The identification of differentially expressed miRNAs between cells infected with virulent and avirulent Mtb can be used to rapidly screen virulent strains to determine appropriate treatments.

Changes in miRNA expression that are induced by Mtb infection may also be caused by other diseases in the body. Therefore, it is necessary to identify miRNAs that distinguish TB from other diseases, improving the accuracy of using miRNAs as biomarkers in the diagnosis of TB. miRNAs in the serum can distinguish patients with lung cancer, TB, and pneumonia. miR-21 and miR-155 are notably increased in the serum of patients with lung cancer and pneumonia compared to normal controls, and miR-182 is only crucially elevated in the serum of patients with lung cancer (230). One study measured plasma miRNA levels in patients with chronic obstructive pulmonary disease (COPD), asthma, and pulmonary TB. miR-21 and miR-34a are increased in patients with COPD and asthma, whereas miR-206 is decreased. miR-133 decreases in patients with COPD and TB and can be used to distinguish these patients from those with asthma (231). Crohn’s disease (CD) and intestinal TB have similar features and insensitive diagnostic tools, which makes their identification extremely difficult, but the use of miRNAs as biomarkers can solve this problem. The plasma miR-375-3p concentration is higher in patients with ITB than in patients with CD, whereas higher miR-375-3p expression is observed in the tissues of patients with CD (232). The clinical treatment and prognosis of tuberculosis pleural effusion (TPE) and malignant pleural effusion (MPE) are completely different, and effective biomarkers can quickly diagnose patients and enable them to receive effective treatment. miR-195-5p, miR-182-5p, and miR-34a-5p expression levels are much higher in patients with MPE than in patients with TPE, and they may be potential biomarkers for MPE diagnosis (233).

### Signs of active versus latent TB

**Figure 4:**
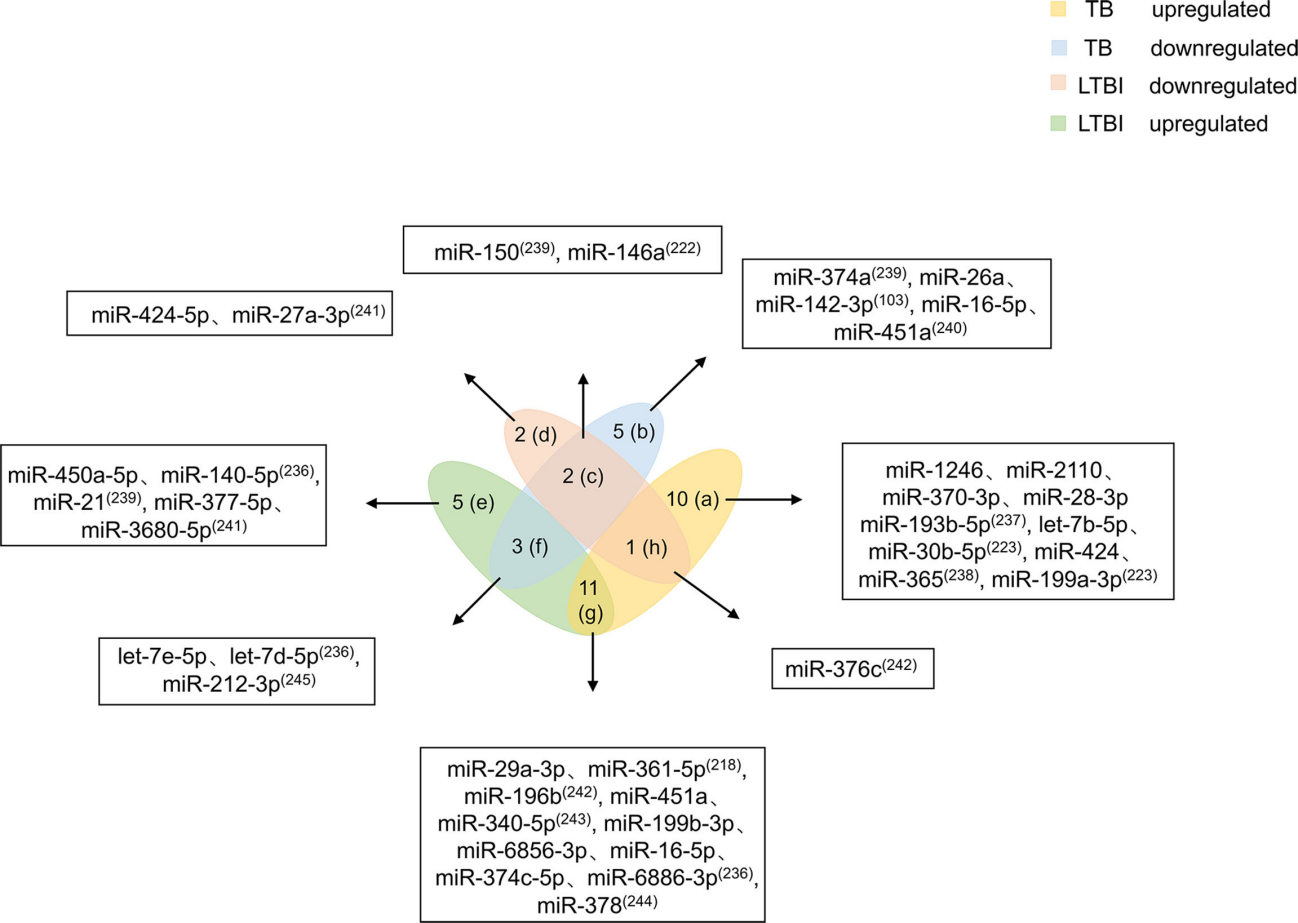
Differential expression of miRNAs in TB and LTBI. Differentially expressed miRNAs were divided into eight categories **(A–H)**. miRNAs in the a region are only upregulated in TB patients. miRNAs in the b region are reduced only in TB patients. miRNAs in the c region diminish in TB and LTBI. miRNAs in the d region are decreased in patients with LTBI. miRNAs in the e region are increased in patients with LTBI. miRNAs in the f region are elevated in LTBI but downregulated in TB. miRNAs in the g region are both increased. miRNA in the h region is higher in TB, but decreases in LTBI. miRNAs in the **(C, F–H)** regions should be focused on, and miRNAs in the f and h regions have higher potential for biomarkers.

Mtb infection does not mean people will get sick. LTBI refers to host infection with Mtb without TB symptoms. When the host is infected with Mtb, the body’s immune cells cannot clear the pathogen in a timely manner. However, due to immune-mediated control of the infection, the patient does not experience the clinical symptoms of TB (6). Studies have shown that people with LTBI have a 5%-15% risk of developing active TB (234). Existing screening methods, such as interferon-γ release assays and tuberculin skin tests, are mainly used to diagnose Mtb infection based on a response of the patient to Mtb antigenic stimulation. However, these tests cannot differentiate active TB from LTBI, and both tests have lower accuracy in immunocompromised patients (235). Because no test has been found that effectively distinguishes LTBI from active TB, it is necessary to identify differentially expressed biomarkers between the two conditions. This review summarizes 39 miRNAs related to LTBI and TB (**Figure 4**).

Based on miRNA upregulation and downregulation in LTBI and TB, 39 miRNAs are divided into eight categories. Ten miRNAs (miR-1246, miR-2110, miR-370-3p, miR-28-3p, miR-193b-5p, let-7b-5p, miR-30b-5p, miR-424, miR-365, and 199a-3p) are upregulated only in TB patients (223, 236–238). Five miRNAs (miR-374a, miR-26a, miR-142-3p, miR-16-5p, and miR-451a) are downregulated only in TB patients (103, 239, 240). miR-424-5p and miR-27a-3p expression is decreased in patients with LTBI (241). Elevated expression levels of five miRNAs (miR-450a-5p, miR-140-5p, miR-21, miR-377-5p, and miR-3680-5p) are observed in patients with LTBI (236, 239, 241). The miRNAs that are mentioned above and only upregulated or downregulated in LTBI and TB patients cannot be used as biomarkers alone. More attention should be given to miRNAs that are at the intersection of expression in patients with LTBI and TB. miR-150 and miR-146a expression is decreased in patients with TB and LTBI (222, 239). Of course, there are miRNAs (miR-29a-3p, miR-361-5p, miR-196b, miR-451a, miR-340-5p, miR-199b-3p, miR-6856-3p, miR-16-5p, miR-374c-5p, miR-6886-3p, and miR-378) whose expression is increased in both patients with TB and LTBI (218, 223, 242–244). The number of miRNAs with elevated expression in LTBI and TB is higher, which may be caused by the host immune response to pathogen infection. In addition, four miRNAs are differentially expressed in patients with TB and LTBI. let-7e-5p, let-7d-5p, and miR-212-3p are upregulated in patients with LTBI but downregulated in patients with TB (236, 245). miR-376c is higher in patients with TB, but it is decreased in patients with LTBI (242).

### Potential and challenges associated with the use of miRNAs as biomarkers for TB

As a new type of biomarker, miRNAs have promising application prospects (1). miRNAs are very stable. As short-chain noncoding RNA molecules with only 22 nucleotides, miRNAs are extremely stable in serum, plasma, urine, and other samples (246, 247). miRNAs maintain good stability even after prolonged storage and freeze−thaw cycles (248). This property is advantageous for the use of miRNAs as potential biomarkers for clinical disease (2). A wide range of miRNA sources are available. miRNAs can be easily obtained from cells, extracellular fluids, and body fluids. Changes in the expression of miRNAs can be rapidly measured by quantitative polymerase chain reaction (3). miRNAs can serve as biomarkers of TB in multiple ways. Commonly used TB detection methods can effectively identify infected patients. However, ideal results from other perspectives, such as distinguishing LTBI from TB, identifying drug-resistant patients, and determining the prognosis after treatment, cannot be obtained. miRNAs can distinguish TB patients from healthy controls, TB patients from LTBI patients, treated patients from untreated patients, and TB patients from patients with other diseases. miRNAs can even help to identify the drug resistance and virulence of Mtb (4). In this study, six miRNAs (miR-29, miR-361, miR-146, miR-26, miR-199, and miR-16) were identified that can distinguish between healthy individuals and TB, TB and LTBI patients before and after treatment. miR-212, miR-378, miR-196, miR-365, and miR-30 are differentially expressed in healthy individuals, TB patients, and LTBI patients, and miR-30 can also identify virulent strains. miR-148, miR-346, miR-155, miR-99, and miR-125 can be used as biomarkers to distinguish healthy individuals, TB patients, and TB patients after treatment, and miR-125 can also identify virulent strains. miR-223, miR-197, let-7, and miR-320 can not only indicate infected patients but also identify drug-resistant patients (5). When searching PubMed with the keywords “micro-RNA” and “Biomarker,” nearly 30,000 results were found. miRNAs not only can be used for the diagnosis of TB but also have excellent application prospects in aging-related diseases and cancer.

The use of miRNAs as biomarkers also faces many challenges. First, many miRNAs that can be used as biomarkers have identified, but the mechanisms of most miRNAs remain unclear. Second, there are differences in miRNA expression patterns between individuals. Similarly, differences in the age and sex of subjects causes different results in the use of miRNAs as biomarkers. In addition, the strength of the immune system can also affect the results. Suitable miRNA biomarkers should be stable enough to be useful in this role under dissimilar conditions. Finally, miRNAs should be disease specific, and reliable biomarkers should be able to differentiate TB, LTBI, and other respiratory diseases. To solve these problems, the following solutions can be adopted in future research. First, miRNAs that have the potential to be used as biomarkers should be examined, and the specific mechanisms by which these miRNAs function should be thoroughly explored. Next, the combination of multiple miRNAs can mitigate the differences that are observed between individuals and improve the accuracy of the detection results. Finally, standardized miRNA expression data can be established to differentiate TB, LTBI, and other respiratory diseases.

## Conclusion and future perspectives

TB, which is a highly contagious disease, is difficult to control or completely cure with traditional treatment and detection methods in a timely manner. It is important to develop effective tests and treatments for TB. Accumulating evidence suggests that miRNAs are of considerable importance for host immunity. Some miRNAs positively regulate the immune response to clear pathogens in response to Mtb infection. However, Mtb can upregulate the expression of certain miRNAs and suppress the expression of immune-related genes to evade clearance mechanisms. This suggests that we can target miRNAs to enhance the function of the immune system to treat TB.

In addition, we still need to pay attention to some limitations in the current research. First, a large number of studies have focused on how miRNAs regulate inflammation, autophagy, and apoptosis while ignoring the important role of miRNAs in regulating metabolism. Mtb continuously exchanges substances and energy within host cells to maintain its own growth and reproduction. The identification of miRNAs that specifically inhibit the metabolic pathway of Mtb may be possible to fundamentally cure tuberculosis. Second, although many miRNAs have been shown to target certain genes to regulate anti-Mtb immune responses, their specific molecular mechanisms remain unclear. Can one microRNA target multiple genes and can these genes interact with each other during Mtb infection? Could this interaction play a greater role in amplifying antibacterial effects or would they counteract each other? Finally, certain miRNAs may play opposite roles in innate and acquired immune responses. At present, studies on the roles of miRNAs in antibacterial immunity have mainly focused on innate or acquired immunity, but these branches of the immune response function as a whole and should not be separated. For some miRNAs with potential antibacterial effects, the functions and specific molecular mechanisms of their target genes should be explored in representative innate and acquired immune cells.

At present, there are some tests that can detect tuberculosis quickly and effectively. However, these traditional methods are less effective in distinguishing among TB patients, LTBI patients and healthy individuals. LTBI has a strong incubation period, and if not detected and treated with preventive drugs, such patients will develop active TB. Compared with traditional methods, detecting differentially expressed miRNAs in patients is more efficient and rapid. Only real-time fluorescence quantitative polymerase chain reaction is required to obtain the results. In addition, the combination of multiple miRNAs can improve the accuracy of detection results. As treatment progresses, miRNA expression patterns will sensitively change, which is beneficial for doctors when administering targeted treatment to patients. However, there are no systematic and standardized quantitative methods for measuring miRNA expression for clinical diagnosis. This greatly affects the accuracy and application of miRNAs as biomarkers for TB diagnosis. Therefore, in the future, miRNAs can be used as targets to advance research on the treatment and diagnosis of TB in order to cure this disease that plagues humans as soon as possible.

## Author contributions

LW, XL, and HW drafted the initial manuscript with feedback from all authors. YX, BF, DG and MZ all gave their comments and suggestions to the manuscript. XL and HW reviewed and modified the manuscript. All authors read and approved the final manuscript.

## Funding

This work was supported by the National Natural Science Foundation of China (No. 81970008, 82000020), the Fundamental Research Funds for the Central Universities (No. 2022CDJXY-004, 2019CDYGZD009 and 2020CDJYGRH-1005), Natural Science Foundation of Chongqing, China (cstc2020jcyj-msxmX0460) and Chongqing Talents: Exceptional Young Talents Project (No. cstc2021ycjh-bgzxm0099). The funders had no role in study design, data collection and analysis, decision to publish, or preparation of the manuscript.

## Acknowledgments

The authors would like to thank Dr. Tao Li (Wuhan University) and Dr. Rui Wang (Sun Yat-sen University) for providing helpful comments and critical suggestions.

## Conflict of interest

The authors declare that the research was conducted in the absence of any commercial or financial relationships that could be construed as a potential conflict of interest.

## Publisher’s note

All claims expressed in this article are solely those of the authors and do not necessarily represent those of their affiliated organizations, or those of the publisher, the editors and the reviewers. Any product that may be evaluated in this article, or claim that may be made by its manufacturer, is not guaranteed or endorsed by the publisher.

## Abbreviations

TB, Tuberculosis; Mtb, Mycobacterium tuberculosis; LTBI, Latent tuberculosis infection; IL, Interleukin; BMDM, Bone marrow-derived macrophage; IFN-γ, Interferon-γ; ABCA1, ATP binding cassette subfamily A member 1; THP-1, Transformed human mononuclear cell line; TLRs, Toll-like receptors; DCs, Dendritic cells; SHIP-1, Src homologous 2-inositol phosphatase-1; AMPK, AMP-activated protein kinase; LC3, Microtubule-associated protein light chain 3; ER, Endoplasmic reticulum; STAT3, Signal transducer and activator of transcription 3; UVRAG, UV radiation resistance associated gene; ATM, Ataxia telangiectasia mutated; PKA, cAMP dependent protein kinase; FOXO3, Forkhead box O3; JNK2, c-Jun N-terminal kinase 2; COPD, Chronic obstructive pulmonary disease; TPE, Tuberculosis pleural effusion; COVID-19, Coronavirus disease 2019; miRNAs, micro-RNAs; ATP, Adenosine triphosphate; Bcl-2, B-cell lymphoma/leukemia-2; PFK-M, Phosphor-fructokinase, muscle; SREBP, Sterol regulatory element binding protein; HDL, High density lipoprotein; NF-κB, Nuclear factor NF-kappa-B; TNF-α, Tumor necrosis factor-alpha NK, Natural killer; NLRP3, NLR family pyrin domain containing 3; ATG, Autophagy-related genes; ULK1, Unc-51 like autophagy activating kinase 1; Mcl-1, Myeloid cell leukemia sequence 1; TWEAK, TNF superfamily member 12; MyD88, Myeloid differentiation primary response gene 88; DRAM2, DNA damage regulated autophagy modulator 2; PBMCs, Peripheral blood mononuclear cells; MDMs, Monocyte derived macrophages; NEK6, NIMA related kinase 6; CD, Crohn’s disease; MPE, Malignant pleural effusion.

## Glossary

**Table:**

TB	Tuberculosis
Mtb	Mycobacterium tuberculosis
LTBI	Latent tuberculosis infection
IL	Interleukin
BMDM	Bone marrow-derived macrophage
IFN-γ	Interferon-γ
ABCA1	ATP binding cassette subfamily A member 1
THP-1	Transformed human mononuclear cell line
TLRs	Toll-like receptors
DCs	Dendritic cells
SHIP-1	Src homologous 2-inositol phosphatase-1
AMPK	AMP-activated protein kinase
LC3	Microtubule-associated protein light chain 3
ER	Endoplasmic reticulum
STAT3	Signal transducer and activator of transcription 3
UVRAG	UV radiation resistance associated gene
ATM	Ataxia telangiectasia mutated
PKA	cAMP dependent protein kinase
FOXO3	Forkhead box O3
JNK2	c-Jun N-terminal kinase 2
COPD	Chronic obstructive pulmonary disease
TPE	Tuberculosis pleural effusion
COVID-19	Coronavirus disease 2019
miRNAs	micro-RNAs
ATP	Adenosine triphosphate
Bcl-2	B-cell lymphoma/leukemia-2
PFK-M	Phosphor-fructokinase muscle
SREBP	Sterol regulatory element binding protein
HDL	High density lipoprotein
NF-kB	Nuclear factor NF-kappa-B
TNF-α	Tumor necrosis factor-alpha
NK	Natural killer
NLRP3	NLR family pyrin domain containing 3
ATG	Autophagy-related genes
ULK1	Unc-51 like autophagy activating kinase 1
Mcl-1	Myeloid cell leukemia sequence 1
TWEAK	TNF superfamily member 12
MyD88	Myeloid differentiation primary response gene 88
DRAM2	DNA damage regulated autophagy modulator 2
PBMCs	Peripheral blood mononuclear cells
MDMs	Monocyte derived macrophages
NEK6	NIMA related kinase 6
CD	Crohn’s disease
MPE	Malignant pleural effusion
